# Viro-immunological evaluation in an immunocompromised patient with long-lasting SARS-CoV-2 infection

**DOI:** 10.1080/22221751.2022.2045877

**Published:** 2022-03-10

**Authors:** A. Mancon, A. Rizzo, D. Mileto, S. Grosso, A. Foschi, M. Cutrera, A. Capetti, I. Faggion, A. Anselmo, A. Monte, S. Fillo, G. Rizzardini, M. R. Gismondo, V. Micheli

**Affiliations:** a Laboratory of Clinical Microbiology, Virology and Bioemergencies, ASST Fatebenefratelli Sacco – University of Milan, Milan, Italy; b Department of Infectious Diseases, ASST Fatebenefratelli Sacco, Milan, Italy; c Scientific Department Army Medical Center, Rome, Italy; d L. Sacco Department of Biomedical and Clinical Sciences, University of Milan, Milan, Italy

**Keywords:** SARS-CoV-2, viral evolution, seroconversion, IFN-γ, immunosuppression

## Letter

Severe acute respiratory syndrome coronavirus 2 (SARS-CoV-2) infection is usually self-limiting. However, several evidences suggest the possibility of prolonged viral shedding and re-infection, giving rise to doubt on transmission potential and immunity protection [1,2]. Here we report the case of SARS-CoV-2 evolution and re-emergence three months after recovery presenting prolonged absence of humoral response and high production of virus-induced IFN-γ.

In March 2020 a woman whit history of follicular lymphoma successfully treated with obinutuzumab plus cyclophosphamide, doxorubicin, vincristine, and prednisone (G-CHOP), was hospitalized with pneumonia: the Real-Time PCR (Allplex™ 2019-nCoV Assay; Table S1**)** performed on nasopharyngeal swab (NPS) revealed SARS-CoV-2 infection. Given persistence of fever, headache, cough and bilateral bronchial thickening, the subject was moved to Sacco Hospital Infectious Disease ward and then discharged on April 28th, after two negative NPSs and conditions amelioration **(**Figure S1**)**.

However, due to fever reappearance, the patient required a new hospitalization on May 6th. The chest x-ray showed interstitial inflammation and Computer Tomography (CT) scan with contrast highlighted ground glass opacities in chest and hilar and mesenteric adenopathy, compatible with the previous lymphoma, while NPSs resulted negative for SARS-CoV-2 RNA. A ciprofloxacin 250 mg *bis in die* treatment was administrated due to urinary tract infection, switched to amoxicillin/clavulanic acid considering *E. feacalis* isolation and antibiotic sensitivity test. Moreover, bronchoalveolar lavage fluid (BALF) was positive for both Aspergillus antigen and SARS-CoV-2 RNA and a voriconazole plus piperacillin/tazobactam regimen was started. Since chest CT showed COVID-19 pulmonary picture, IL-6 concentration and SARS-CoV-2 RNA in plasma were evaluated, as indicators of negative prognosis: viral RNA was not present, while a high IL-6 concentration was found (56 ng/L, reference value <7 ng/L); in addition, both anti-SARS-CoV-2 IgG and IgM tested negative (iFlash-SARS-COV-2 IgM/IgG Antibody Test**;** Table S1), also considering a B cells count of 0.001 10^3^ cells/µL (Table S2). After improvement of clinical conditions and two negative NPSs the patient was discharged on 11th June.

In September the woman had another ER access, with fever and cough and chest x-ray showing right lung upper lobe involvement and lobular thickening, requiring hospitalization. The new NPS resulted positive for SARS-CoV-2 RNA, while no seroconversion was found (IgG <3.8 AU/mL, LIAISON^®^ SARS-CoV-2 S1/S2; Table S1), even in December and January (Figure S1).

**Figure 1.**
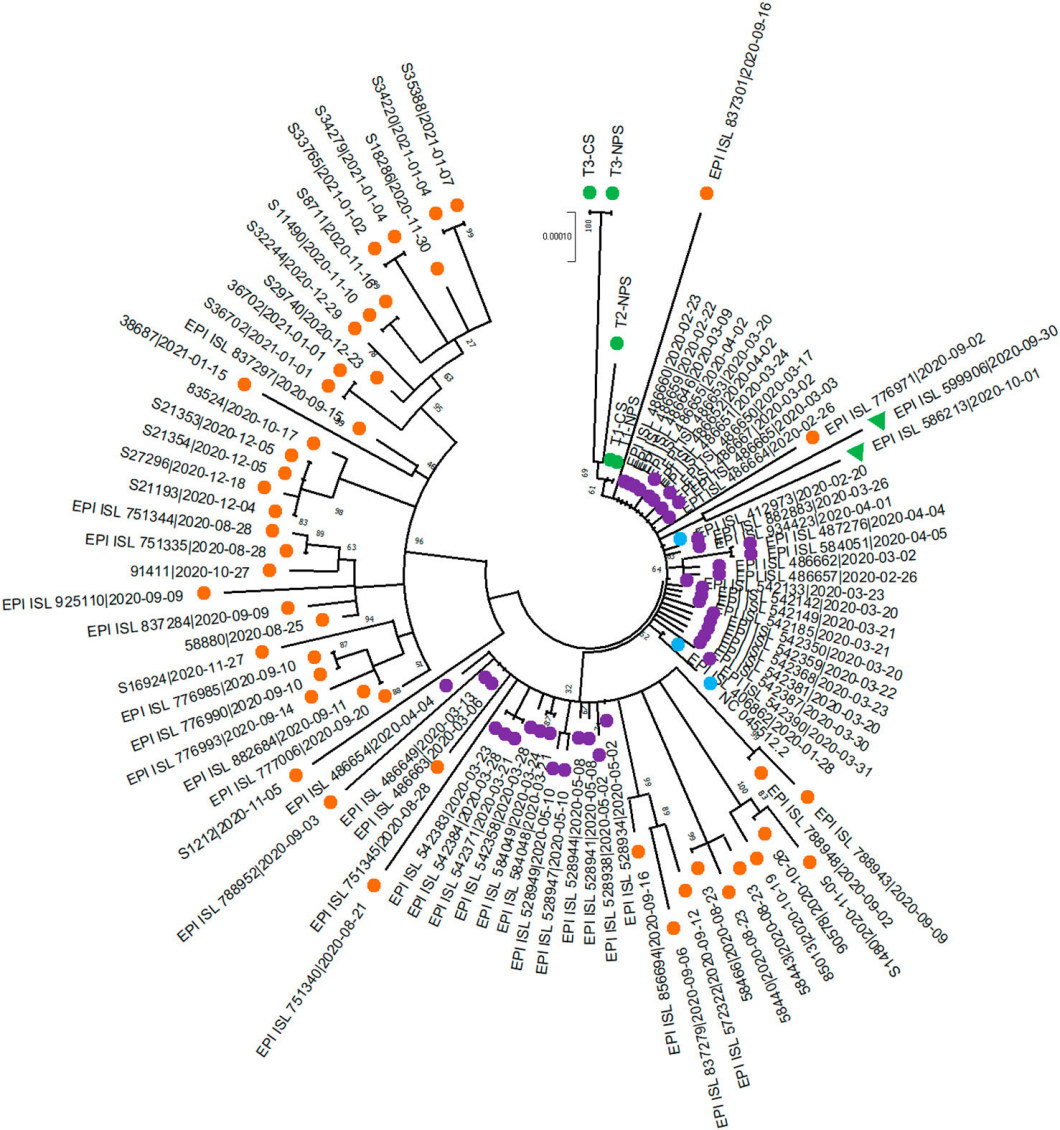
Phylogenetic tree showing relationship between patient’s sequences and other viral isolates. Phylogenetic analysis revealed a close relationship between patient’s viral sequences (green circles), forming a small cluster with no further sequence: purple circles refer to first epidemic wave isolates, as T1-NPS and T2-BALF, while orange to the second one as T3-NPS. No correlation was found with B.1.235 virus (green triangles), SARS-CoV-2 reference sequence, nor German and Italian patients 1 (light-blue circles).

Given the possibility of re-infection, further investigations were conducted. Viral isolation in Vero E6 cells was attempted: after 72 h of cultivation, three out of five samples showed a cytopathic effect (CPE) and a diminution in Ct values was observed performing the Real-time PCR on cell culture supernatants (Table S3). The March (T1-NPS) and September (T3-NPS) NPSs, BALF (T2-BALF) and viral supernatants (T1-CS and T3-CS) were used for Whole Genome Sequencing (WGS) with CleanPlex^®^ SARS-CoV-2 Panel (Paragon Genomics, Inc. USA), mapping reads against the Wuhan reference genome (GenBank accession number: NC_045512.2) and assigning lineages with Pangolin COVID-19 Lineage Assigner software (https://cov-lineages.org/pangolin.html): T1-NPS and T2-BALF isolates were attributed to B.1 lineage, while T3-NPS to B.1.235 one. Comparing T1, T2, and T3 sequences, accumulation and parallel loss of mutations were detected, as exemplified by S-gene: D614G was conserved among all viruses, I1179 T and I1210 T were present in T2-NPS only, while A222 V in T3-NPS (Table S4). Occurrence of re-infection was thus supposed and a phylogenetic analysis was performed: an alignment was created including patient’s sequences, isolates of first and second epidemic waves, two B.1.235 and SARS-CoV-2 reference (Table S5); genetic correlation was inferred in MEGA X (statistical method: Maximum Likelihood, model: Hasegawa-Kishino-Yano, Bootstrap replicates: 1000), returning a patient’s sequences self-standing clade with no other sequence, neither B.1.235 ones supposed to cluster with T3-NPS (Figure 1).

Phylogenetic and mutational data suggested that the first infection virus persisted and gradually evolved, probably favoured by long-lasting immunodepression, even after vaccination. In fact, on February 2021 the patient received BNT162b2 (Comirnaty) and vaccine response was evaluated measuring serum anti-S1/S2 antibodies (Abs) on March 12th, April 30th and June 23rd, and anti-RBD Abs (LIAISON^®^ SARS-CoV-2 TrimericS IgG; Table S1) on November 5th. Specific CD4^+^ and CD4^+^/CD8^+^ T-cell mediated response was evaluated too, quantifying IFN-γ release (Quanti-FERON SARs-CoV-2; Table S1), after *in-vitro* T-cell stimulation with viral peptide pools. Absence of anti-S1/S2 and anti-RBD Abs was detected, while strong specific T-cells mediated response was present: IFN-γ values were Ag1 = 2.32 IU/mL, Ag2 = 2.76 IU/mL and Ag3 = 4.6 IU/mL (Figure S1) [3–5].

## Discussion

Here we presented the case of a patient experiencing a second SARS-CoV-2 infection, after complete remission and virus undetectability, suggesting possible reinfection favoured by protective Abs absence. Such hypothesis was supported by the detection of different lineages and quite divergent mutational patterns between the viruses sustaining the two infections. However, the phylogenetic analysis revealed that viral sequences clustered together excluding any other one, even those belonging to the B.1.235 lineage, suggesting viral persistence and intra-host evolution. Such phenomenon had been already described for well-studied pathogens, such as HIV, HCV, HCMV [6–8] and viral quasi-species production and compartmentalization had been described for SARS-CoV, MERS-CoV, and SARS-CoV-2 [9,10]. The wide expression in many tissues of SARS-CoV-2 receptor Angiotensin-Converting Enzyme 2 (ACE-2) would allow the virus to potentially replicate, hide, and persist in multiple organs, especially if lack of immune response occurs. Obinutuzumab, used for follicular lymphoma treatment, induces a B cell depletion, that could last for several months even after discontinuation, preventing antibodies production and allowing virus replication, as also demonstrated by CPE in September cell culture [11]. The effects of persistence and evolution can be multiple. The continuous stimulation of CD4 and CD8 cells could have induced sustained anti-SARS-CoV-2 IFN-γ production: the values here reported were considerably elevated when compared to those obtained in infection-naive subjects 90 days after second vaccine dose administration; however, the T-cells response seemed inadequate for virus clearance in neutralizing Abs absence [5]. Viral replication under multifactorial stimulation (i.e.: concomitant infections, comorbidities therapy), could lead to intra-host evolution and emergence of new virus, impairing antibodies neutralization, as evidenced in HIV viremic patients [12,13]; moreover, some researchers speculated that this mechanism could drive SARS-CoV-2 variants generation, given that Omicron one emerged in a population with a high proportion of untreated HIV patients [14].

In conclusion, the present case underlines how immune-depressed patients can experience long-lasting SARS-CoV-2 infections, that could result in the emergence of viruses with new and potentially challenging characteristics, as exemplified by the so-called Variants of Concern; moreover, joint evaluation of antibodies and specific T-cell responses is fundamental to study and manage infection in this setting of patients: persistent absence of neutralizing Abs is likely and it may be not balanced out by T-cells response.

## Supplementary Material

Supplemental Material

## References

[1] Cevik M, Tate M, Lloyd O, et al. SARS-CoV-2, SARS-CoV-1 and MERS-CoV viral load dynamics, duration of viral shedding and infectiousness: a living systematic review and meta-analysis. medRxiv 2020.07.25.20162107. 10.1016/S2666-5247(20)30172-5 PMC7837230 33521734

[2] Mileto D, Foschi A, Mancon A, et al. A case of extremely prolonged viral shedding: could cell cultures be a diagnostic tool to drive the COVID-19 patients discharge? Int J Infect Dis. 2020;104:631–633. doi:10.1016/j.ijid.2020.11.161. 33227514 PMC7679112

[3] Van Praet JT, Vandecasteele S, De Roo A, et al. Humoral and cellular immunogenicity of the BNT162b2 messenger RNA coronavirus disease 2019 vaccine in nursing home residents. Clin Infect Dis. 2021 Dec 6;73(11):2145–2147. doi:10.1093/cid/ciab300. 33825869 PMC8083580

[4] Tychala A, Meletis G, Katsimpourlia E, et al. Evaluation of the QuantiFERON SARS-CoV-2 assay to assess cellular immunogenicity of the BNT162b2 mRNA COVID-19 vaccine in individuals with low and high humoral response. Hum Vaccin Immunother. 2021 Oct 29: 1–2. doi:10.1080/21645515.2021.1991710. PMC8567290 34714711

[5] Mileto D, Fenizia C, Cutrera M, et al. SARS-CoV-2 mRNA vaccine BNT162b2 triggers a consistent cross-variant humoral and cellular response. Emerg Microbes Infect. 2021 Dec;10(1):2235–2243. doi:10.1080/22221751.2021.2004866. 34749573 PMC8648019

[6] Giatsou E, Abdi B, Plu I, et al. Ultradeep sequencing reveals HIV-1 diversity and resistance compartmentalization during HIV-encephalopathy. AIDS. 2020;34(11):1609–1614. doi:10.1097/QAD.0000000000002616. 32701585

[7] Sorbo MC, Carioti L, Bellocchi MC, et al. HCV resistance compartmentalization within tumoral and non-tumoral liver in transplanted patients with hepatocellular carcinoma. Liver Int. 2019;39(10):1986–1998. doi:10.1111/liv.14168. 31172639

[8] Pang J, Slyker JA, Roy S, et al. Mixed cytomegalovirus genotypes in HIV-positive mothers show compartmentalization and distinct patterns of transmission to infants. Elife. 2020;9:e63199. doi:10.7554/eLife.63199. 33382036 PMC7806273

[9] Xu D, Zhang Z, Wang FS. SARS-associated coronavirus quasispecies in individual patients. N Engl J Med. 2004;350(13):1366–1367. doi:10.1056/NEJMc032421. PMID: 15044654. 15044654

[10] Rueca M, Bartolini B, Gruber C, et al. Compartmentalized replication of SARS-Cov-2 in upper vs. lower respiratory tract assessed by whole genome quasispecies analysis. Microorganisms; 8(9):1302. doi:10.3390/microorganisms8091302. PMC7563410 32858978

[11] Redfield RR, Jordan SC, Busque S, et al. Safety, pharmacokinetics, and pharmacodynamic activity of obinutuzumab, a type 2 anti-CD20 monoclonal antibody for the desensitization of candidates for renal transplant. Am J Transplant. 2019;19(11):3035–3045. doi:10.1111/ajt.15514. 31257724 PMC6899639

[12] Weigang S, Fuchs J, Zimmer G, et al. Within-host evolution of SARS-CoV-2 in an immunosuppressed COVID-19 patient as a source of immune escape variants. Nat Commun. 2021;12:6405. doi:10.1038/s41467-021-26602-3. 34737266 PMC8568958

[13] Hoffman SA, Costales C, Sahoo MK, et al. SARS-CoV-2 neutralization resistance mutations in patient with HIV/AIDS, California, USA. Emerg Infect Dis. 2021;27(10):2720–2723. doi:10.3201/eid2710.211461. 34296992 PMC8462335

[14] Kupferschmidt K. Where did “weird” Omicron come from? Science. 2021 Dec 3;374(6572):1179. doi:10.1126/science.acx9738. 34855502

