# Supplementary figures and images for: Viro-immunological evaluation in an immunocompromised patient with long-lasting SARS-CoV-2 infection
Source: Emerg Microbes Infect. 2022 Mar 10;11(1):786–9. doi: 10.1080/22221751.2022.2045877 (PMC8920372; doi:10.1080/22221751.2022.2045877)

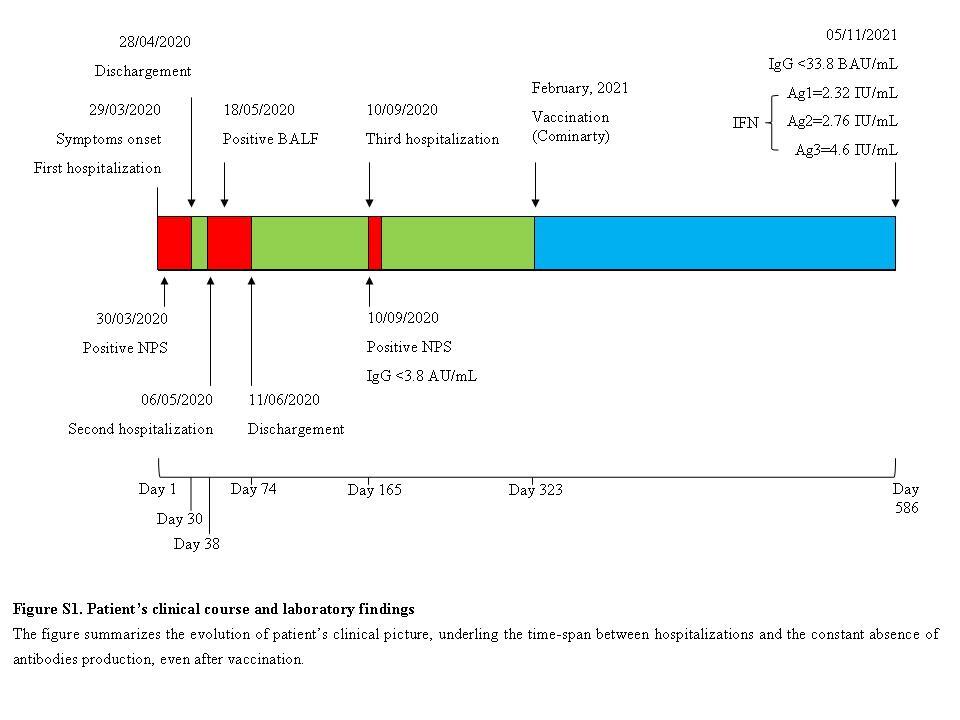

Supplement: Supplemental Material [file TEMI_A_2045877_SM0569.zip › Suppl files/Figure S1.jpg]
