# Supplementary material for: Viro-immunological evaluation in an immunocompromised patient with long-lasting SARS-CoV-2 infection
Source: Emerg Microbes Infect. 2022 Mar 10;11(1):786–9. doi: 10.1080/22221751.2022.2045877 (PMC8920372; doi:10.1080/22221751.2022.2045877)
Supplement: Supplemental Material [file TEMI_A_2045877_SM0569.zip › Suppl files/Table S1.doc]

| **Assay** | **Targets** | **Limits** | **Company** |
| --- | --- | --- | --- |
| Allplex™ SARS-CoV-2 Assay (RT-PCR) | E, N and RdRp | not appliable | Seegene Inc., Republic of Korea |
| LIAISON^®^ SARS-CoV-2 S1/S2 IgG (CLIA) | S1 and S2 proteins | < 12 AU/mL = negative | DiaSorin, Saluggia, Italy |
|  |  | 12-15 AU/mL = borderline |  |
|  |  | ≥ 15 AU/mL = positive |  |
| CleanPlex^®^ SARS-CoV-2 Panel (NGS) | Whole genome | not appliable | Paragon Genomics, Inc. USA |
| LIAISON^®^ SARS-CoV-2 TrimericS IgG (CLIA) | Trimeric S protein | < 33.8 BAU/mL | DiaSorin, Saluggia, Italy |
|  |  | ≥ 33.8 BAU/mL |  |
| iFlash-SARS-CoV-2 IgM and IgG (CLIA) | N and S proteins | < 10 AU/mL = negative | Shenzen YHLO Biotech Co., Ltd., |
|  |  | ≥ 10 AU/mL = positive | Shenzen, China |
| Quanti-FERON^®^ SARS-CoV-2 (ELISA) | CD4 IFN (S1 stimulation) | < 0.15 AU/mL = negative | QIAGEN, Hilden, Germany |
|  | CD4+CD8 IFN (S1, S2 stimulation) | ≥ 0.15 AU/mL = positive |  |
|  | CD4+CD8 IFN (M, N, S stimulation) |  |  |

E: Envelope, N: Nucleocapside, ORF: Open Reading Frame, S: Surface protein, UTR: Untranslated region, AA: aminoacide

**Table S1. Molecular and serology assays used to monitor SARS-CoV-2 infection**

Considering subject’s conditions, different tests were used to characterize virus and immune response. In particular, serological assays varied according to availability, implementation and target: iFlash-SARS-CoV-2 IgM and IgG was the first kit introduced in virology laboratory, that was then substituted by LIAISON^®^ SARS-CoV-2 S1/S2 IgG and its new release LIAISON^®^ SARS-CoV-2 TrimericS IgG, designed to detect neutralizing Abs; Quanti-FERON^®^ SARS-CoV-2 is the most recent test, developed on the basis of tuberculosis diagnosis to investigate the possible role of T-cells response in SARS-CoV-2 infection and vaccination.
