# Supplementary material for: Viro-immunological evaluation in an immunocompromised patient with long-lasting SARS-CoV-2 infection
Source: Emerg Microbes Infect. 2022 Mar 10;11(1):786–9. doi: 10.1080/22221751.2022.2045877 (PMC8920372; doi:10.1080/22221751.2022.2045877)
Supplement: Supplemental Material [file TEMI_A_2045877_SM0569.zip › Suppl files/Table S2.doc]

| **Sample collection date** | **Assay** | **Result** | **Unit** | **Reference limits** |
| --- | --- | --- | --- | --- |
| 11/05/2020 | B cells (CD19+) | 0,0 | % | 6,0 - 19,0 |
|  | B cells (CD19+) | 0,0 | x10^3^/µL | 14,000 - 5,000 |
|  | T cells CD4+ (CD4+CD3+) | 48,8 | % | 34,0 - 60,0 |
|  | T cells CD4+ (CD4+CD3+) | 0,894 | x10^3^/µL | 6,000 - 15,000 |
|  | T cells CD8+ (CD8+CD3+) | 44,1 | % | 13,0 - 35,0 |
|  | T cells CD8+ (CD8+CD3+) | 0,808 | x10^3^/µL | 22,000 - 87,000 |
|  | Total T cells (CD3+) | 94,2 | % | 64,0 - 83,0 |
|  | Total T cells (CD3+) | 1,703 | x10^3^/µL | 11,000 - 24,000 |
|  | CD4/CD8 Ratio | 1,11 |  |  |
| 20/05/2020 | B cells (CD19+) | 0,1 | % | 6,0 - 19,0 |
|  | B cells (CD19+) | 0,001 | x10^3^/µL | 14,000 - 5,000 |
|  | T cells CD4+ (CD4+CD3+) | 44,4 | % | 34,0 - 60,0 |
|  | T cells CD4+ (CD4+CD3+) | 0,512 | x10^3^/µL | 6,000 - 15,000 |
|  | T cells CD8+ (CD8+CD3+) | 48,5 | % | 13,0 - 35,0 |
|  | T cells CD8+ (CD8+CD3+) | 0,558 | x10^3^/µL | 22,000 - 87,000 |
|  | Total T cells (CD3+) | 94,1 | % | 64,0 - 83,0 |
|  | Total T cells (CD3+) | 1,115 | x10^3^/µL | 11,000 - 24,000 |
|  | CD4/CD8 Ratio | 0,92 |  |  |

**Table S2. Results of B and T cells determination**

The complete absence of antibodies and the detection of IFN-production are strictly related to lymphocyte population, that were influenced by lymphoma treatment. As reported in the table, T cells were present, both as CD4+ and CD8+ subpopulation, while B cells production was still suppressed; unfortunately, no further determination were available, thus making impossible to evaluate immune status overtime, especially after vaccination.
