# Supplementary material for: Viro-immunological evaluation in an immunocompromised patient with long-lasting SARS-CoV-2 infection
Source: Emerg Microbes Infect. 2022 Mar 10;11(1):786–9. doi: 10.1080/22221751.2022.2045877 (PMC8920372; doi:10.1080/22221751.2022.2045877)
Supplement: Supplemental Material [file TEMI_A_2045877_SM0569.zip › Suppl files/Table S3.doc]

| **SAMPLE DATE** | **MATERIAL** | **RT-PCR** | **RT-PCR Ct** | **CPE** | **VC RT-PCR Ct** |
| --- | --- | --- | --- | --- | --- |
| 31/03/2020 | NPS | P | 30 | P | 14.2 |
| 07/04/2020 | NPS | P | 15 | P | 12 |
| 24/04/2020 | NPS | N | NA | NA | NA |
| 27/04/2020 | NPS | N | NA | NA | NA |
| 06/05/2020 | NPS | N | NA | NA | NA |
| 07/05/2020 | NPS | N | NA | NA | NA |
| 18/05/2020 | BALF | P | 29 | N | 32.46 |
| 27/05/2020 | NPS | N | NA | NA | NA |
| 28/05/2020 | PLASMA | N | NA | NA | NA |
| 09/06/2020 | NPS | N | NA | NA | NA |
| 10/06/2020 | NPS | N | NA | NA | NA |
| 10/09/2020 | NPS | P | 26 | P | 13.28 |
| 21/09/2020 | NPS | LP | 35 | N | 37.15 |
| 09/12/2020 | NPS | N | NA | NA | NA |

CPE: Cytopathic Effect; VC: Viral Culture; NPS: nasopharyngeal swabs; BALF: bronchoalveolar lavage fluid; P: positive; N: negative; LP: low positive (Ct >32); NA: not available.

**Table S3. Comparison between NPSs RT-PCR Ct values, Cytopathic Effect detection and Viral Culture RT-PCR Ct values.**

The table summarizes the data obtained testing patient’s samples with RT-PCR and viral culture. It clearly appears how the virus disappeared from upper respiratory tract in late April, remaining undetectable until the symptoms reappearance in September: such evidences suggested the possibility of re-infection, then excluded thanks to sequence analysis.
