# Supplementary material for: Viro-immunological evaluation in an immunocompromised patient with long-lasting SARS-CoV-2 infection
Source: Emerg Microbes Infect. 2022 Mar 10;11(1):786–9. doi: 10.1080/22221751.2022.2045877 (PMC8920372; doi:10.1080/22221751.2022.2045877)
Supplement: Supplemental Material [file TEMI_A_2045877_SM0569.zip › Suppl files/Table S4.doc]

|  | **Genome position** | | | | | | | | | | | | | | | | | | | | | | | | | | | |
| --- | --- | --- | --- | --- | --- | --- | --- | --- | --- | --- | --- | --- | --- | --- | --- | --- | --- | --- | --- | --- | --- | --- | --- | --- | --- | --- | --- | --- |
|  | **5'UTR** | | **ORF1a** | | | | | | | | | | **ORF1b** | | | | | | | **S** | | | | | **E** | | **ORF7b** | **N** |
|  | 187 | 241 | 683 | 2707 | 3037 | 4423 | 4893 | 5628 | 8394 | 9438 | 11898 | 13458 | 14408 | 14724 | 15336 | 18063 | 19170 | 20052 | 21133 | 21742 | 22227 | 23403 | 25098 | 25191 | 26270 | 26333 | 27758 | 28657 |
| **T1** | A>G | C>T | - | - | C>T | - | - | - | - | - | - | - | C>T | - | - | - | - | - | - | - | - | A>G | - | - | - | - | - | - |
| **T2** | A>G | C>T | - | - | C>T | - | - | - | - | C>T | A>G | - | C>T | - | - | - | - | A>T | - | - | - | A>G | T>C | T>C | C>T | C>T | - | - |
| **T3** | A>G | C>T | C>T | A>G | C>T | C>A | C>T | C>T | C>T | - | - | C>T | C>T | C>T | T>A | T>C | C>T | - | C>A | C>T | C>T | A>G | - | - | - | C>T | del | C>T |
| **AA Changes** |  |  |  |  |  |  | T1543I | T1788M | A2710V | T3058I | Q3878R | S4398L | P4715L |  |  |  |  |  | Q6957K | A222V |  | D614G | I1179T | I1210T | T9I | T30I |  |  |

E: Envelope, N: Nucleocapside, ORF: Open Reading Frame, S: Surface protein, UTR: Untranslated region, AA: aminoacide

**Table S4. Virus genomic mutations at the three different time-points**

The three viral sequences shares five mutations, while many other are present only at T2 and T3: in particular, the latter shows a higher number of nucleotides substitutions, that could be related to persistence
