# Supplementary material for: Viro-immunological evaluation in an immunocompromised patient with long-lasting SARS-CoV-2 infection
Source: Emerg Microbes Infect. 2022 Mar 10;11(1):786–9. doi: 10.1080/22221751.2022.2045877 (PMC8920372; doi:10.1080/22221751.2022.2045877)
Supplement: Supplemental Material [file TEMI_A_2045877_SM0569.zip › Suppl files/Table S5.doc]

**Sequences GISAID ID**

hCoV-19/Italy/LOM-UniMI-1/2020|EPI_ISL_487276|2020-04-04

hCoV-19/Italy/LOM-UniMI-L137/2020|EPI_ISL_542133|2020-03-23

hCoV-19/Italy/LOM-UniMI-L147/2020|EPI_ISL_542142|2020-03-20

hCoV-19/Italy/LOM-UniMI-L154/2020|EPI_ISL_542149|2020-03-21

hCoV-19/Italy/LOM-UniMI-L195/2020|EPI_ISL_542185|2020-03-21

hCoV-19/Italy/LOM-UniMI-L431/2020|EPI_ISL_542350|2020-03-20

hCoV-19/Italy/LOM-UniMI-L439/2020|EPI_ISL_542358|2020-03-28

hCoV-19/Italy/LOM-UniMI-L440/2020|EPI_ISL_542359|2020-03-22

hCoV-19/Italy/LOM-UniMI-L450/2020|EPI_ISL_542368|2020-03-23

hCoV-19/Italy/LOM-UniMI-L453/2020|EPI_ISL_542371|2020-03-21

hCoV-19/Italy/LOM-UniMI-L465/2020|EPI_ISL_542381|2020-03-20

hCoV-19/Italy/LOM-UniMI-L470/2020|EPI_ISL_542383|2020-03-23

hCoV-19/Italy/LOM-UniMI-L471/2020|EPI_ISL_542384|2020-03-28

hCoV-19/Italy/LOM-UniMI-L474/2020|EPI_ISL_542387|2020-03-30

hCoV-19/Italy/LOM-UniMI-L477/2020|EPI_ISL_542390|2020-03-31

hCoV-19/Italy/LOM-UniMI-ICCS1/2020|EPI_ISL_584048|2020-03-21

hCoV-19/Italy/LOM-UniMI-ICCS2/2020|EPI_ISL_584049|2020-03-24

hCoV-19/Italy/LOM-UniMI-vir1/2020|EPI_ISL_584051|2020-04-05

hCoV-19/Italy/LOM-CR02/2020|EPI_ISL_882883|2020-03-26

hCoV-19/Italy/LOM-CRGS-2/2020|EPI_ISL_934423|2020-04-01

hCoV-19/Italy/LOM-IZSGC-TE30933/2020|EPI_ISL_528934|2020-05-02

hCoV-19/Italy/LOM-IZSGC-TE31751/2020|EPI_ISL_528938|2020-05-02

hCoV-19/Italy/LOM-IZSGC-TE35432/2020|EPI_ISL_528941|2020-05-08

hCoV-19/Italy/LOM-IZSGC-TE35532/2020|EPI_ISL_528944|2020-05-08

hCoV-19/Italy/LOM-IZSGC-TE38811/2020|EPI_ISL_528947|2020-05-10

hCoV-19/Italy/LOM-IZSGC-TE38845/2020|EPI_ISL_528949|2020-05-10

hCoV-19/Italy/CAM-IZSM-166/2020|EPI_ISL_572322|2020-09-12

hCoV-19/Italy/VEN-UniVR-11/2020|EPI_ISL_751335|2020-08-28

hCoV-19/Italy/VEN-UniVR-19/2020|EPI_ISL_751340|2020-08-21

hCoV-19/Italy/VEN-UniVR-25/2020|EPI_ISL_751344|2020-08-28

hCoV-19/Italy/VEN-UniVR-27/2020|EPI_ISL_751345|2020-08-28

hCoV-19/Italy/CAM-TIGEM-324/2020|EPI_ISL_776971|2020-09-02

hCoV-19/Italy/CAM-TIGEM-334/2020|EPI_ISL_776985|2020-09-10

hCoV-19/Italy/CAM-TIGEM-306/2020|EPI_ISL_776990|2020-09-10

hCoV-19/Italy/CAM-TIGEM-360/2020|EPI_ISL_776993|2020-09-14

hCoV-19/Italy/CAM-TIGEM-376/2020|EPI_ISL_777006|2020-09-20

hCoV-19/Italy/APU-PODV-15917816/2020|EPI_ISL_788943|2020-09-09

hCoV-19/Italy/APU-PODV-15956923/2020|EPI_ISL_788948|2020-09-02

hCoV-19/Italy/APU-PODV-15961609/2020|EPI_ISL_788952|2020-09-03

hCoV-19/Italy/CAM-TIGEM-1027/2020|EPI_ISL_837279|2020-09-06

hCoV-19/Italy/CAM-TIGEM-999/2020|EPI_ISL_837284|2020-09-09

hCoV-19/Italy/CAM-TIGEM-1006/2020|EPI_ISL_837297|2020-09-15

hCoV-19/Italy/CAM-TIGEM-1007/2020|EPI_ISL_837301|2020-09-16

hCoV-19/Italy/CAM-COTUGNO-54438/2020|EPI_ISL_856694|2020-09-16

hCoV-19/Italy/CAM-CRGS-233/2020|EPI_ISL_882684|2020-09-11

hCoV-19/Italy/CAM-CRGS-261/2020|EPI_ISL_925110|2020-09-09

hCoV-19/Wales/PHWC-47D95A/2020|EPI_ISL_586213|2020-10-01

hCoV-19/England/MILK-A05FB9/2020|EPI_ISL_599906|2020-09-30

hCoV-19/Italy/LOM-ASST-CDG1/2020|EPI_ISL_412973|2020-02-20

hCoV-19/Germany/BY-ChVir-929/2020|EPI_ISL_406862|2020-01-28

hCoV-19/Italy/LOM-ASST-2/2020|EPI_ISL_486646|2020-03-09

hCoV-19/Italy/LOM-ASST-10/2020|EPI_ISL_486647|2020-03-02

hCoV-19/Italy/LOM-ASST-11/2020|EPI_ISL_486648|2020-03-02

hCoV-19/Italy/LOM-ASST-12/2020|EPI_ISL_486649|2020-03-13

hCoV-19/Italy/LOM-ASST-13/2020|EPI_ISL_486650|2020-03-17

hCoV-19/Italy/LOM-ASST-14/2020|EPI_ISL_486651|2020-03-24

hCoV-19/Italy/LOM-ASST-15/2020|EPI_ISL_486652|2020-04-02

hCoV-19/Italy/LOM-ASST-16/2020|EPI_ISL_486653|2020-03-20

hCoV-19/Italy/LOM-ASST-17/2020|EPI_ISL_486654|2020-04-04

hCoV-19/Italy/LOM-ASST-18/2020|EPI_ISL_486655|2020-04-02

hCoV-19/Italy/LOM-ASST-19/2020|EPI_ISL_486656|2020-02-20

hCoV-19/Italy/LOM-ASST-20/2020|EPI_ISL_486657|2020-02-26

hCoV-19/Italy/LOM-ASST-3/2020|EPI_ISL_486659|2020-02-22

hCoV-19/Italy/LOM-ASST-4/2020|EPI_ISL_486660|2020-02-23

hCoV-19/Italy/LOM-ASST-5/2020|EPI_ISL_486661|2020-03-02

hCoV-19/Italy/LOM-ASST-6/2020|EPI_ISL_486662|2020-03-02

hCoV-19/Italy/LOM-ASST-7/2020|EPI_ISL_486663|2020-03-06

hCoV-19/Italy/LOM-ASST-8/2020|EPI_ISL_486664|2020-02-26

hCoV-19/Italy/LOM-AMVRC-9/2020|EPI_ISL_486665|2020-03-03

**Table S5. References for SARS-CoV-2 GISAID sequences used in phylogenetic analysis.**

The sequences retrieved from GISAID and used in this paper are reported in table 2. In particular: sequences from EPI_ISL_486646 to EPI_ISL_486665 were generated in Sacco Laboratory of Clinical Microbiology, Virology and Bioemergencies in March-April 2020; EPI_ISL_412973 corresponds to Italian Patient 1 virus; EPI_ISL_586213 and EPI_ISL_599906 are lineage B.1.235 from England and Wales; EPI_ISL_406862 is the sequence of first German autochthonous patient, the first described case of local transmission in Europe. Other sequences included in this study are undergoing GISAID submission.
